# Iron Uptake Analysis in a Set of Clinical Isolates of *Pseudomonas putida*

**DOI:** 10.3389/fmicb.2016.02100

**Published:** 2016-12-27

**Authors:** Lázaro Molina, Valérie A. Geoffroy, Ana Segura, Zulema Udaondo, Juan-Luis Ramos

**Affiliations:** ^1^Environmental Protection Department, Estación Experimental del Zaidín, Consejo Superior de Investigaciones CientíficasGranada, Spain; ^2^Centre National de la Recherche Scientifique, UMR 7242, Université de Strasbourg, (ESBS)Illkirch, France

**Keywords:** siderophores, pyoverdine, iron, clinical strains, *Pseudomonas putida*

## Abstract

*Pseudomonas putida* strains are frequent inhabitants of soil and aquatic niches and they are occasionally isolated from hospital environments. As the available iron sources in human tissues, edaphic, and aquatic niches are different, we have analyzed iron-uptake related genes in different *P. putida* strains that were isolated from all these environments. We found that these isolates can be grouped into different clades according to the genetics of siderophore biosynthesis and recycling. The pyoverdine locus of the six *P. putida* clinical isolates that have so far been completely sequenced, are not closely related; three strains (*P. putida* HB13667, HB3267, and NBRC14164T) are grouped in Clade I and the other three in Clade II, suggesting possible different origins and evolution. In one clinical strain, *P. putida* HB4184, the production of siderophores is induced under high osmolarity conditions. The pyoverdine locus in this strain is closely related to that of strain *P. putida* HB001 which was isolated from sandy shore soil of the Yellow Sea in Korean marine sand, suggesting their possible origin, and evolution. The acquisition of two unique TonB-dependent transporters for xenosiderophore acquisition, similar to those existing in the opportunistic pathogen *P. aeruginosa* PAO, is an interesting adaptation trait of the clinical strain *P. putida* H8234 that may confer adaptive advantages under low iron availability conditions.

## Introduction

*Pseudomonas putida* is a species that is widely distributed in the environment, being particularly abundant in rhizospheric, and bulk soil (Molina et al., 2000; Fernández et al., 2013; García-Salamanca et al., 2013), as well as in fresh water communities (Maki et al., 1994; Li et al., 2012; Udaondo et al., 2012). Recently, a few *P. putida* strains have been isolated from hospital environments (von Graevenitz and Weinstein, 1971; Galli et al., 1992; Yoshino et al., 2011; Molina et al., 2013, 2014, 2016). The ubiquitous character of this microorganism has been linked to its broad metabolic potential, its ability to produce a large variety of secondary metabolites that may enable colonization of different environments and a complex array of sigma factors and two-component regulatory systems that allow a fast adaptation to environmental changes (Matilla et al., 2011; Udaondo et al., 2012, 2013, 2016; Planchamp et al., 2015).

Iron has been recognized as a limiting nutrient and consequently, microbes have to assure iron sensing and uptake in each of the niches they colonize. The main mechanism for capturing iron is through high affinity of siderophores (Payne, 1980; Schalk and Guillon, 2013; Gasser et al., 2015). *P. putida* produces a main siderophore that has been previously identified as pyoverdine (Matthijs et al., 2009). From a chemical point of view, pyoverdine has a structure made up of three distinct elements: a quinoline-1-carboxylic acid moiety that contains a chromophore responsible for green fluorescence (a feature that is highly conserved in all the described pyoverdines); a dicarboxylic acid or its monoamide, bound as an amide to the 5-amino group of the chromophore; and a peptidic chain comprised of 6–14 amino acids bound to the carboxyl group of the quinoline (Meyer, 2000; Visca et al., 2007; Cornelis, 2010). Different types of pyoverdine have been found at the species level; these are often visualized by different band patterns in isoelectric focusing (IEF) gels, a method termed siderotyping that has been used as a taxonomic tool (Koedam et al., 1994; Fuchs et al., 2001; Meyer et al., 2007, 2008; Ye et al., 2013). In *P. putida* strains, siderotyping correlates with the phylogeny of genes involved in pyoverdine production, and it was found to be useful in distinguishing different *P. putida* strains (Meyer et al., 2007; Ye et al., 2013). Pyoverdine binds iron with high affinity and the complex is recognized by an outer membrane receptor named FpvA. The FpvA protein has been used as a phylogenetic marker because it shows high divergence and substantial intratype variation, and it has been shown that this variability is not generated by recombination (Smith et al., 2005).

In addition to self-produced pyoverdines, *P. putida* strains are able to take up iron through the acquisition of siderophores produced by other microorganisms (xenosiderophores); for this acquisition, *P. putida* utilizes a number of TonB-dependent membrane transporters. The large number of these transporters found in *P. putida* genomes correlates with their capacity for adaptation to changing environments and enhanced niche colonization (Martínez-Bueno et al., 2002; Cornelis, 2010; Nader et al., 2011).

The aim of this study was to study the iron uptake systems (pyoverdine locus and iron-related TonB-dependent transporters) in a set of clinical *P. putida* strains to try to understand the impact of the iron uptake systems on the capacity of clinical strains to colonize and survive in humans tissues. To this end, we used annotated genomes from *P. putida* strains available in the NCBI data base (http://www.ncbi.nlm.nih.gov/genome/?term = putida, May 2016), that includes six clinical *P. putida* isolates, to carry out *in silico* studies of the pyoverdine locus and the TonB-dependent transporters. To support the bioinformatic studies we have used four available *P. putida* clinical strains. The four strains were isolated at the Hospital of Besançon (France). *P. putida* HB13667 and H8234 were isolated from the blood of two in-patients presenting general bacteremia (Molina et al., 2013, 2016); *P. putida* HB4184 was isolated from sputum of a cystic-fibrosis patient (Molina et al., 2016); and *P. putida* HB3267 which exhibits an antibiotic multi-resistant phenotype and was isolated from the blood of a deceased in-patient (Molina et al., 2014, 2016).

Our analyses indicate that *P. putida* strains can be classified into five clearly defined clades on the basis of the homology of proteins related with pyoverdin production and that the six clinical isolates belong to two different clades suggesting that they have at least two different phylogenic origins. Strain *P. putida* H4184 shares many common siderophore-related elements with one rhizobacteria strain (*P. putida* B001) isolated from high osmolarity environments; accordingly, we have demonstrated that this strain produces a large amount of siderophores under high osmolarity conditions.

Interestingly, the clinical strain *P. putida* H8234 presented two unique TonB-related transporters, similar to those of the opportunistic pathogen *P. aeruginosa* PAO; these transporters can confer selective advantages against other *P. putida* strains in environments with a low iron content.

## Materials and methods

### Bioinformatic analysis

All the strains used in this study are listed in Table 1. Their sequences are available in the GenBank data base. Genome comparisons to determine protein homology, common genes, and genomic organization were performed using the RAST software (Aziz et al., 2008) and BLASTp.

**Table 1:** **Strains used in this study and their main characteristics related to pyoverdine biosynthesis and uptake**.

		**Strains**	**Origin, properties**	**Loci number**	**Predicted peptide**		**FpvA type**	**Other TonB-transporters**	**References**
Clade I	Subclade A	*P. putida* HB13667	Clinical, blood	2	Asp-Lys-OHAsp-Ser	This study	FpvA-I (Subgroup I)	8	Molina et al., 2016
		*P. putida* HB3267	Clinical, blood	2	Asp-Lys-OHAsp-Ser	Ye et al., 2013		8	Molina et al., 2014
	8	*P. putida* JLR11	Waste water	2	Asp-Lys-OHAsp-Ser	This study			Pascual et al., 2015
	Subclade B	*P. monteilii* SB3101	Waste water	2	Asp-eLys-OHAsp-Ser	This study		8	Bogaerts et al., 2011
		*P. taiwanensis* SJ9	Industrial waste water	2	Asp-eLys-OHAsp-Ser	This study		8	Hong et al., 2016
		*P. putida* S16	Rice rhizosphere	2	Asp-eLys-OHAsp-Ser	Ye et al., 2013		8	Yu et al., 2011
		*P. putida* KG-4	Soil, naphthalene degrader	2	Asp-eLys-OHAsp-Ser	This study		9	Dawar and Aggarwal, 2015
	Subclade C	*P. putida* NBRC14164T	Clinical^*^	2	Asp-eLys-OHAsp-Ser	This study		20	Ohji et al., 2014
		*P. putida* GB-1	Aquatic, manganese oxidizer	2	Asp-eLys-OHAsp-Ser	Ye et al., 2013		24	Wu et al., 2011
Clade II	Subclade A	*P. putida* HB8234	Clinical, Blood	1	Asp-5hOrn-Lys-(Thr/Gly)	This study	FpvA-I (Subgroup II)	19	Molina et al., 2013
		*P. putida* SJ3	Waste water, caprolactam degrader	1	Asp-5hOrn-Lys-(Thr/Gly)	This study		18	Hong et al., 2015
		*P. putida* OUS82	Soil, naphthalene degrader	1	Asp-5hOrn-Lys-(Thr/Gly)	This study		18	Tay et al., 2014
		*P. putida* JCM18798	Clinical^*^	1	Asp-5hOrn-Lys-(Thr/Gly)	This study		19	PRJDB838
	Subclade B	*P. putida* HB4184	Clinical, sputum	1	Asp-5hOrn-Lys-(Thr/Gly)	This study		10	Molina et al., 2016
		*P. putida* B001	Rhizosphere, high osmolarity resistant	1	Asp-5hOrn-Lys-(Thr/Gly)	This study		11	Park et al., 2011
Clade III		*P. putida* KT2440	Rhizosphere	1	Asp-Orn-(OHAsp-Dab)-Gly-Ser-cOHOrn	Ye et al., 2013	FpvB	11	Nelson et al., 2002
		*P. putida* BIRD-1	Rhizosphere	1	Asp-Orn-(OHAsp-Dab)-Gly-Ser	Ye et al., 2013		13	Matilla et al., 2011
					-cOHOrn				
		*P. putida* PCL1760	Rhizosphere	1	Asp-Orn-(OHAsp-Dab)-Gly-Ser-cOHOrn	This study		8	PRJNA289510
		*P. putida* Idaho	n.d., solvent tolerant	1	Asp-Orn-(OHAsp-Dab)-Gly-Ser-cOHOrn	This study		8	Tao et al., 2011
		*P. putida* S12	n.d., solvent tolerant	1	Asp-Orn-(OHAsp-Dab)-Gly-Ser-cOHOrn	Ye et al., 2013		10	Tao et al., 2012
Clade IV		*P. putida* F1	Water, toluene degrader	2	Asp-Orn-Dab-Thr-Gly	Ye et al., 2013	FpvA-III	11	Wu et al., 2011
		*P. putida* DOT-T1E	Waste water, solvent tolerant	3	Asp-Orn-Dab-Thr-Gly	This study		11	Udaondo et al., 2012
		*P. putida* ND6	Waste water, naphthalene degrader	2	Asp-Orn-Dab-Gly-Ser	Ye et al., 2013		11	Li et al., 2012
		*P. putida* TR01	Waste water, triclosan degrader	2	Asp-Orn-Dab-Thr-Gly	Ye et al., 2013		10	Maki et al., 1994
		*P. putida* LS46	Waste water, PHA synthesizer	2	Asp-Orn-Dab-Thr-Gly	Ye et al., 2013		9	Sharma et al., 2012
Clave V		*P. putida* W619	Endophytic	1	Ser-Xxx_OHAsp-Gly-Thr	Ye et al., 2013	Different FpvA	6	Wu et al., 2011
		*P. putida* SQ1	Sediment of a lake	1	Ser-Xxx_OHAsp-Gly-Thr	This study		6	Felux et al., 2015
		*P. putida* ATH-43	n.d., Antarctic environment	1	Ser-Xxx_OHAsp-Gly-Thr	This study		7	PRJNA278654

*Asp, Aspartic acid; OHAsp, threo-β-hydroxy-aspartic acid; Dab, diamino-butanoic acid; Gly, glycine; Lys, Lysine; εLys, Lys linked by its ε-NH_2_; Orn, Ornithine; cOHOrn, δN-hydroxy-ornithine; 5hOrn, 4,5-hydroxy-L-proline; Ser, Serine; Thr, Threonine; Xxx, undetermined*.

n.d not described

* *No isolation location was provided other than the human body*.

Phylogenetic studies of individual proteins were performed using the platform Phylogeny.fr (http://www.phylogeny.fr/) (Dereeper et al., 2008). Phylogenetic studies of complete genomes were performed using Composition Vector Tree (http://tlife.fudan.edu.cn/cvtree/). These phylogenetic studies were carried out as recommended by the user manual (http://tlife.fudan.edu.cn/cvtree/help/index.html) with a K-peptide length of 6, as recommended by the authors for prokaryote organisms (Xu and Hao, 2009).

The pyoverdine peptide chain was predicted using the non-ribosomal peptide synthase substrate predictor NRPSsp (http://nrpssp.com/execute.php), NRPSpredictor2 (http://nrps.informatik.uni-tuebingen.de), and PKS/NRPS programs (http://nrps.igs.umaryland.edu/) for pyoverdine synthetases.

TonB-dependent transporters were identified by selecting TonB annotated proteins from the genomes of the microorganisms used in this study using the RAST Annotation Server (http://rast.nmpdr.org/rast.cgi) and the set of sequences deposited in Genbank (https://www.ncbi.nlm.nih.gov/genbank/). We selected TonB-transporter proteins bearing the characteristic domains of siderophore transporters by application of InterPro (http://www.ebi.ac.uk/interpro/) and Blastp web sites that provide functional analysis. Exclusive TonB-dependent transporters were those presenting < 80% identity in the amino-acid sequence with others present in *P. putida* strains.

### Production of pyoverdine

Cells were pre-grown in M9 minimal media (Abril et al., 1989) with glucose (5 mM) as the sole carbon source. The overnight cultures were washed three times and re-suspended to reach OD_660nm_ of 0.1 in M9-glucose. Salt conditions were adjusted to 0.05 or 0.9% w/v of NaCl in the low and high osmolarity media respectively. When indicated the iron chelator EDDHA was added to reach a final concentration of 20 μM. The visual and spectrophotochemical detection of pyoverdine production was performed as indicated by Bultreys et al. (2001). The production of pyoverdine-type siderophores was visualized as maximum absorption at 365, 375, and 410 nm.

### IEF analyses of pyoverdines

Supernantants of cultures grown with 5 g/L of casamino acids (CAA) supplemented with MgSO_4_ 7H_2_0 (0.25 g/L) and K_2_HPO_4_ (1.18 g/L) were analyzed by IEF according to the method described by Koedam et al. (1994). Pyoverdine was separated in polyacrylamide (5%) gels (125 × 65 × 0.4 mm) containing 2% ampholines (Byolyte 3/10 from Bio-Rad) to develop a pH gradient from 3.5 to 9.3 during electrophoresis in model 111 Mini IEF Cell. Preparation of the gels and electrophoresis conditions were those recommended by the manufacturer. Samples of 1 μl of the 20-fold-concentrated (through lyophilisation) CAA-culture supernatants were loaded. Samples were run at 4°C for 1.5 h at constant power (12W) with voltage from 200 V at the beginning to 1000 V at the end of the electrophoresis. Immediately after the run, the fluorescent bands of pyoverdines were visualized under UV light at 365 nm.

### Siderophore uptake experiments

Siderophore-mediated iron uptake was performed as described by Fuchs et al. (2001) and adapted by Hoegy and Schalk (2014). Briefly, labeled ferrisiderophores were prepared by mixing a 4μl^55^FeCl_3_ 250 μM (Amersham) with 4 μl of a 1 mM pyoverdine solution obtained from the cultures of the different strains. After 30 min of incubation at room temperature, this mixture was diluted with Tris-HCl pH 8 (50 mM) to obtain a 10 μM siderophore-^55^Fe complex.

*Pseudomonas* strains to be used as siderophore uptaking cells were grown in CAA medium for 24 h at 30°C. Then, bacterial cells were washed twice in CAA medium to remove the native siderophore and were re-suspended in Tris-HCl pH 8 (50 mM) to reach an optical density of 1 at 600 nm.

Five microlitres of siderophore-^55^Fe complex were mixed with 500 μl of the cell suspension and incubated for 30 min at 30°C. Then, 100 μl of the labeled bacterial cells were withdrawn and rapidly filtered through 0.45 μm membranes (Whatmann GFB filters). The cells remaining on the filters were thoroughly washed twice with Tris-HCl pH 8 (50 mM) and the radioactivity was measured to determine the amount of labeled iron incorporated during the incubation time Gamma emission was counted using a PerkinElmer gamma counter. Iron uptake of heterologous pyoverdine was expressed in percentage terms compared with the homologous pyoverdine uptake.

### Statistical analyses

Statistics were evaluated with the Statgraphics Plus v.5.1. software (Statistical Graphics Corp., Herndon, VA, United States). Data are means of three independent determinations and were subjected to one- or two-way analysis of variance (ANOVA) followed by Tukey *posthoc* test (*p* ≤ 0.01) when a significant difference was detected.

## Results

### Diversity of pyoverdine loci in *P. putida* strains

**Figure 1:**
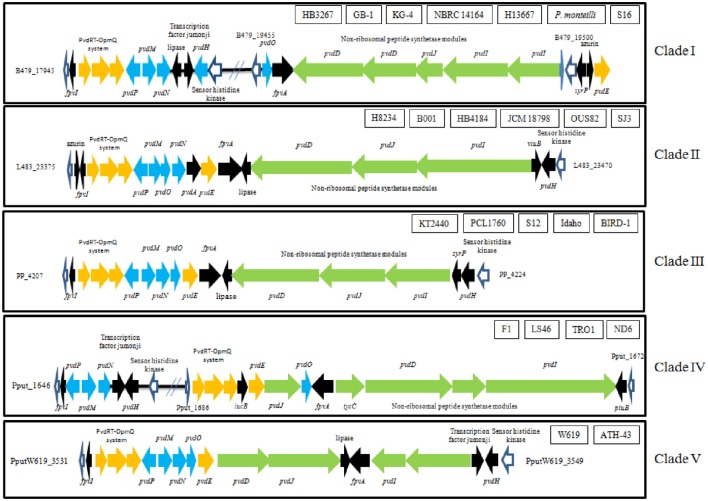
**Organization of the pyoverdine locus in representative ***P. putida*** strains of every clade found**. Green arrows symbolize genes involved in non-ribosomal peptide synthesis modules, yellow/orange arrows indicate genes involved in pyoverdine transport or recycling, blue arrows indicate genes involved in pyoverdine maturation, black arrows other *g*enes involved in pyoverdine biosynthesis and white arrows are genes not directly involved in pyoverdine biosynthesis. Genes without a name, no specific function was found.

The genes which are typically involved in pyoverdine synthesis, transport, and perception are conserved in *P. putida* strains (Figure 1). All the genomes analyzed contain the genes that encode PvdH, PvdP, PvdM, PvdN, PvdO that are involved in the pyoverdine chromophore maturation and PvdD, PvdJ, PvdI which are involved in the synthesis of the peptidic part of the pyoverdine (the non-ribosomal peptide synthetase module). They also contained *pvdE*, a gene that encodes a protein for the export of the pyoverdine precursor peptide to the periplasm, and *pvdR, pvdT*, and *opmQ* and *fvpA*. PvdRT and OpmQ mediate the transport of the newly synthesized pyoverdine across the outer membrane and the recycling of pyoverdines. The FvpA receptor is a TonB-transporter family protein involved in pyoverdine-Fe sensing and internalization (Imperi et al., 2009; Cornelis, 2010; Schalk and Guillon, 2013; Gasser et al., 2015; Chen et al., 2016). *fpvI* that encodes for a RNA polymerase ECF-type sigma factor is also present in all the strains at the same locus. Although all the genes are present in the analyzed genomes, their organization varies depending on the *P. putida* strain (Figure 1).

*P. putida* strains can be grouped into 5 different clades on the basis of the percentage of identity shared by the proteins encoded in the pyoverdine locus (each clade was defined when the overall amino-acid identity was higher than 80%; Tables 1, 2, Figure 1). Strains in Clade I can be classified into two sub-clades, each one sharing an identity higher than 95%. (Tables 1, 2). Subclade A includes the clinical isolates *P. putida* HB13667, HB3267 and the waste-water strain *P. putida* JLR11, (Tables 1, 2). Subclade B included the waste-water strains *P. taiwanensis* SJ9 and *P. monteilii* SB3101; the rhizosphere strain S16 and the soil and naphthalene degrader strain KG-4. The aquatic strain GB-1 and the clinical strain NBRC 14164T form a less homogeneous sub-clade; the amino-acid identity of the pyoverdine locus among the strains of this subclade did not reach 90% (Table 2). In all the strains forming Clade I, the pyoverdine genes are located in two loci in different locations in the chromosome (Table 1, Figure 1).

**Table 2:** **Average of the global identity of the proteins encoded by the pyoverdine genes of the different ***P. putida*** strains**.

**Strain**	**H1**	**H3**	**JL**	***P. t***	***P. m***	**S16**	**KG**	**NB**	**GB**	**H8**	**SJ3**	**OU**	**JC**	**H4**	**B0**	**KT**	**Id**	**BI**	**S12**	**PC**	**F1**	**ND**	**DO**	**TR**	**LS**	**W6**	**SQ1**	**AT**	***P. e***	***P. p***
H13667^*^	**100**	**99.6**	**95.8**	**94.1**	**93.8**	**92.3**	85.7	89.3	82.1	58.2	49.2	53.9	57.6	53.1	53.2	64.8	59.1	59.0	60.8	61.2	56.9	59.1	58.2	60.8	60.8	59.1	62.3	62.6	58.2	38.0
**HB3267**	**98.0**	**100**	**95.6**	**90.7**	**90.6**	88.5	83.5	85.3	87.1	56.4	53.3	56.9	58.9	54.8	54.5	65.0	63.0	62.9	65.1	65.3	60.9	63.3	61.2	64.7	65.8	64.2	63.7	66.2	59.7	40.4
JLR11^*^	**95.8**	**95.1**	**100**	**93.0**	**93.3**	**92.7**	85.2	84.5	81.0	58.1	58.7	58.2	58.5	56.4	57.5	63.1	62.2	65.3	67.8	55.4	62.5	65.2	65.2	64.5	65.2	65.6	63.3	66.3	59.2	36.5
P. t^*^	87.9	88.8	89.2	**100**	**99.5**	**95.4**	**95.8**	80.4	84.3	47.7	48.4	51.2	53.5	58.5	57.3	69.9	70.1	59.7	59.7	62.5	73.0	71.6	72.2	59.4	61.5	57.4	57.1	59.1	61.4	39.1
***P. m***	89.5	89.6	86.4	**96.4**	**100**	**95.4**	**95.8**	87.6	82.4	53.1	50.7	56.7	50.6	47.5	49.5	63.9	59.8	56.5	63.1	61.4	53.0	54.9	59.5	63.3	61.7	58.4	60.3	63.5	56.9	36.8
**S16**	**90.8**	**94.6**	**91.1**	**98.8**	**98.8**	**100**	**95.5**	**90.6**	88.6	53.1	51.8	53.1	53.9	47.4	51.9	62.4	60.5	59.8	62.3	62.3	57.2	62.3	62.8	64.9	64.2	62.5	66.2	64.3	58.7	35.0
KG^*^	**94.4**	**94.4**	**90.0**	**96.0**	**97.7**	**97.7**	**100**	**90.6**	89.4	54.8	55.7	54.7	53.8	53.3	52.1	66.4	67.7	63.4	66.2	66.3	63.9	63.8	64.2	63.2	66.3	63.3	67.3	68.1	63.4	37.4
**NB**	84.5	84.1	80.5	84.5	84.2	83.2	81.9	**100**	**90.7**	53.5	55.4	57.4	54.4	55.4	53.1	61.1	58.5	61.1	61.2	61.4	58.0	59.1	60.1	61.5	62.4	59.6	62.0	62.7	62.4	36.3
**GB-1**	85.9	87.9	84.9	84.1	83.1	82.1	82.1	87.5	**100**	53.6	58.8	63.9	56.1	56.4	54.3	62.8	62.1	62.9	63.0	65.1	60.7	61.1	61.6	63.3	63.8	62.0	68.4	65.9	62.2	41.2
**H8234**	60.0	60.3	62.3	61.5	60.2	59.5	60.5	64.8	63.6	**100**	**99.7**	**96.9**	**96.6**	86.3	86.3	58.8	55.7	58.5	58.8	59.2	58.3	58.7	58.4	58.1	58.6	57.6	57.8	57.7	57.5	42.0
SJ3^*^	51.4	51.5	60.2	57.3	51.8	56.1	56.4	53.5	51.8	**95.2**	**100**	**95.2**	**99.3**	83.1	82.9	50.24	51.3	54.2	54.4	57.0	50.7	53.5	53.6	52.5	52.9	50.5	54.2	54.3	53.1	44.3
OUS82^*^	59.0	58.8	61.5	60.4	59.3	57.8	58.3	63.2	61.7	**99.1**	**99.7**	**100**	**96.0**	86.4	86.3	57.4	53.1	57.2	57.2	56.8	57.2	57.3	57.3	56.5	56.8	56.3	55.9	56.7	56.8	39.8
JC^*^	45.2	45.0	48.1	45.5	45.0	43.1	44.6	45.8	45.5	**92.5**	**96.7**	**96.7**	**100**	81.2	81.6	43.2	51.3	53.1	54.9	53.9	43.5	50.3	53.1	52.2	53.9	44.9	53.3	52.5	52.2	35.2
HB4184^*^	57.1	57.0	59.1	59.1	57.2	56.7	57.8	58.2	57.2	86.0	87.7	87.0	87.9	**100**	**99.3**	55.1	52.2	55.0	55.5	55.4	54.4	55.4	54.9	56.0	55.4	55.8	56.0	56.2	55.6	39.3
B001^*^	58.1	59.3	59.6	60.5	58.4	58.9	59.0	60.0	59.9	85.3	87.9	87.3	88.0	**99.3**	**100**	56.7	54.3	57.8	57.0	57.3	57.1	56.8	56.9	57.2	57.0	56.5	54.9	56.4	56.8	42.1
**KT2440**	72.9	73.2	69.4	61.5	73.0	72.8	72.8	73.0	73.3	57.6	60.1	58.1	61.9	57.7	56.7	**100**	**99.4**	**99.3**	**99.1**	**98.5**	65.7	65.7	66.0	68.7	65.6	66.5	69.2	69.5	64.9	43.8
Idaho^*^	68.6	68.6	68.2	69.7	68.9	68.9	68.4	68.1	68.9	53.1	57.7	53.4	57.9	54.1	53.5	**99.4**	**100**	**99.3**	**99.0**	**98.8**	63.5	63.4	63.6	64.2	64.3	66.5	66.7	66.3	66.1	39.2
**BIRD-1**	66.4	66.6	62.8	67.7	66.7	66.3	66.1	66.3	66.6	49.5	53.5	50.2	49.2	49.9	49.7	**99.3**	**99.3**	**100**	**99.4**	**99.2**	58.1	58.0	58.3	61.2	61.0	60.2	62.9	63.3	59.1	36.2
S12^*^	64.4	66.7	62.8	62.7	66.6	66.3	66.0	66.5	66.7	49.9	53.4	50.2	49.2	49.9	49.6	**99.2**	**99.1**	**99.4**	**100**	**99.8**	58.2	58.0	58.3	61.2	61.0	61.5	62.9	63.2	59.1	36.2
PCL1760^*^	65.4	65.2	61.0	65.7	61.9	61.6	61.6	62.3	62.5	49.5	54.0	50.6	49.1	50.3	49.3	**98.9**	**96.0**	**99.2**	**99.8**	**100**	54.8	57.0	57.2	59.8	60.3	57.6	60.3	61.8	57.6	38.9
**F1**	65.3	63.5	66.6	67.1	64.9	64.2	64.5	66.0	66.0	53.2	58.5	52.6	55.7	54.0	51.9	60.5	60.1	60.3	61.9	60.9	**100**	**99.5**	**99.2**	**99.0**	**96.7**	63.8	64.4	63.6	62.5	41.0
**ND6**	60.1	60.7	61.9	64.5	59.7	61.7	60.0	61.5	60.0	48.5	54.8	51.1	53.2	51.6	51.2	58.0	58.1	55.9	55.9	55.8	**99.5**	**100**	**99.3**	**96.8**	**99.1**	60.4	64.1	63.4	59.6	36.5
**DOT-T1E**	66.0	69.6	64.2	67.8	68.5	68.1	66.6	69.0	70.2	56.9	57.6	52.5	56.6	58.3	52.4	59.2	62.6	58.0	58.0	60.5	**99.1**	**99.4**	**100**	**97.1**	**99.2**	61.2	61.1	61.5	58.1	35.5
TRO1^*^	66.0	66.1	65.2	65.9	65.8	64.7	65.8	66.9	67.1	52.6	54.4	52.7	51.6	53.7	51.5	60.6	61.0	61.4	61.4	61.2	**98.9**	**99.3**	**99.4**	**100**	**99.3**	64.8	64.3	63.2	58.4	34.0
LS46^*^	65.8	66.8	67.1	67.2	65.1	64.3	63.6	67.5	67.4	46.6	56.6	49.5	53.5	54.1	52.3	60.9	61.2	62.6	62.6	60.8	**98.8**	**99.0**	**99.1**	**99.1**	**100**	61.1	64.1	68.3	60.1	35.4
**W619**	68.1	68.2	69.1	68.3	67.9	67.2	67.0	68.1	69.7	52.1	54.4	57.8	56.7	52.4	52.2	62.2	64.2	64.0	64.0	64.0	65.7	65.8	66.3	64.5	64.7	**100**	**98.8**	**98.5**	67.3	46.4
SQ1^*^	66.2	66.1	66.4	64.2	64.6	65.2	66.3	65.8	66.1	52.2	54.1	50.3	50.1	53.3	51.2	62.3	61.2	62.7	61.8	62.2	64.6	64.8	65.0	63.0	61.2	**98.9**	**100**	**98.6**	65.6	47.1
ATH-43^*^	65.2	64.9	68.1	67.3	64.8	64.3	65.8	66.2	67.7	50.2	53.9	51.5	51.1	51.2	50.5	60.5	62.3	62.3	62.2	62.6	64.1	64.6	65.4	63.0	63.2	**98.5**	**98.6**	**100**	66.4	39.1

*H1-H13667. H3- HB3267. JL-JLR11. P.t- P. taiwanensis. P.m- P. monteilli. GB-GB-1. NB-NBRC 14164. KG-KG-4. H8-H8234.OU- OUS82. JC- JCM 18798. H4- HB4184. B0- B001. KT-KT2440. Id-Idaho. BI-BIRD-1. PC-PCL1760. ND-ND-6. DO-DOT-T1E. TR-TRO1. LS- LS46. W6-W619. AT- ATH-43. P.e- P. entomophila. P.p- P. plecoglossicida. Numbers represent the % of identity in protein identity. In bold values over 90%. Highlighted in yellow Clade I strains; in green, Clade II strains; in blue Clade III strains; in red Clade IV strains and in orange Clade V strains (strains considered to belong to the same clade reached values of identity over 80%). Dark tonalities correspond to values over 95%. In the first column*,

* *strains with not closed genome; in red, strains having the same pyoverdine locus as those referred to in Figure 1, in black, strains where the contig structure does not permit the visualization of the pyoverdine locus*.

Clade II can also be divided into two subclades (Tables 1, 2). The first one includes the clinical strains *P. putida* H8234 and JCM 18798; *P. putida* SJ3, a waste-water and caprolactam degrader strain, with the soil isolate and naphthalene degrader *P. putida* OUS82 (Tables 1, 2). Overall amino-acid identity amongst the pyoverdine locus of the four strains within the subclade is higher than 95%. The second subclade includes the clinical strain *P. putida* HB4184 and the rhizobacterium B001 (Table 2). In all the strains forming Clade II, the pyoverdine genes are located in a single locus in the chromosome (Table 1, Figure 1).

Clade III and IV are very homogeneous (Tables 1, 2). Clade III comprises strains that showed more than 98% identity in the pyoverdine-related proteins. As in Clade II, the pyoverdine genes are located in a single locus in all the strains forming Clade III, Table 1, Figure 1). Clade IV, includes strains with an overall identity >96%, and comprises only water isolates, such as the toluene degrading strains *P. putida* F1 and DOT-T1E, the naphthalene degrader ND6, the triclosan degrader TR01, and the polyhydroxyalkanoate synthesizer LS46 (Tables 1, 2). In all these strains the pyoverdine genes are located in two different loci (Table 1, Figure 1), with the exception of *P. putida* DOT-T1E that presents three loci associated with pyoverdine synthesis (Table 1).

The endophitic strain W619, with strain SQ1 isolated from lake sediment and the Antarctic isolate ATH-43 are found in Clade V (Tables 1, 2). In Clade V, the pyoverdine genes are located in a single locus in the chromosome (Table 1, Figure 1).

Two bacteria, traditionally considered close relatives of the *P. putida* group (Molina et al., 2016), were included in this study; the entomopathogen *P. entomophila* L48 (Vodovar et al., 2006), and the fish pathogen *P. plecoglossicida* DSM 15088 (Nishimori et al., 2000). None of them presented an identity higher than 68% at this locus when compared to the rest of the *P. putida* strains (Table 2), indicating the different evolution of these strains in terms of siderophore production and perception.

Bioinformatic prediction of the composition of the peptide sequences of different pyoverdines from several fluorescent *Pseudomonas* was performed by Ye et al. (2013). This study supports our clade classification. Furthermore, we have predicted the pyoverdine peptide sequences of the strains not previously included in Ye et al. (2013) and our results show that all the strains included in a clade have the same peptide sequence (Table 1). The consensus sequence for the pyoverdine peptide obtained for Clade II strains (Table 1) is Asp-5hOrn-Lys-Thr/Gly- a peptide that was not previously predicted by Ye et al. (2013) and that is clearly different from the sequences of other clades.

**Figure 2:**
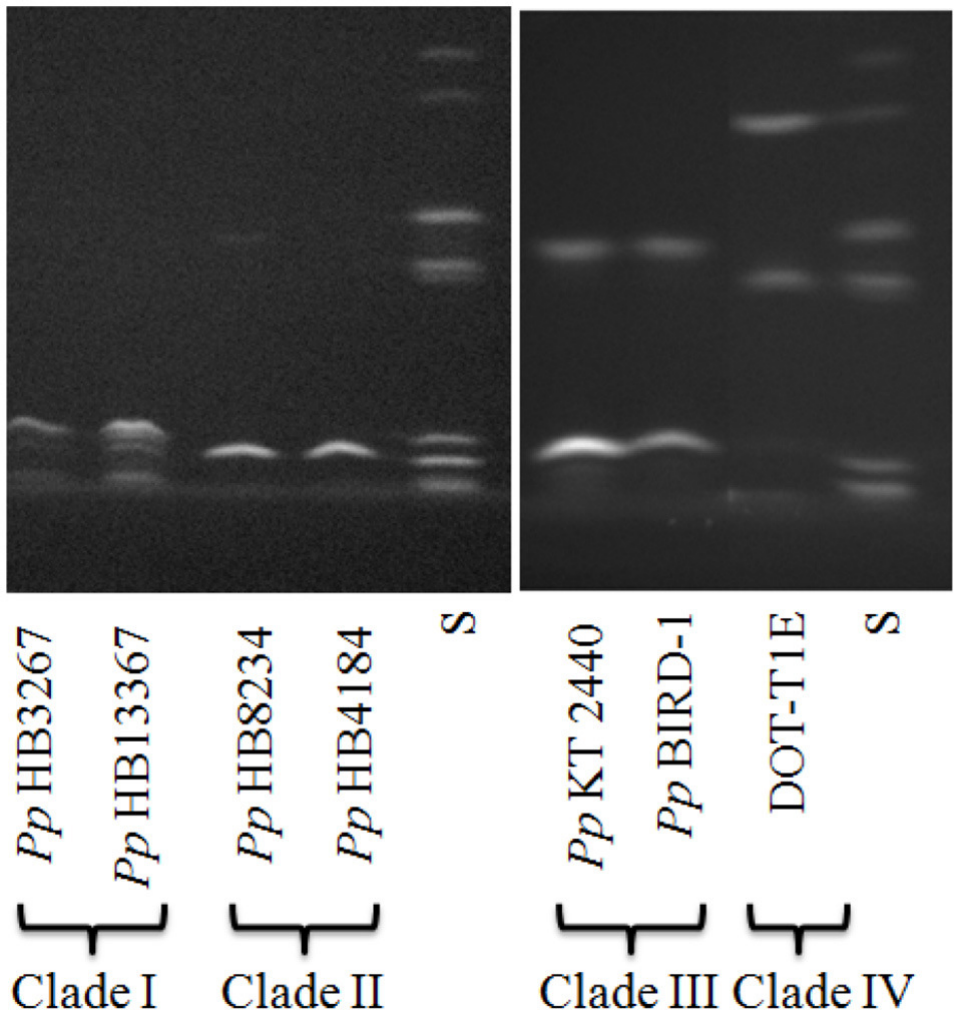
**IEF profiles of pyoverdines synthesized by the different ***P. putida*** strains**. S corresponds to the molecular weight standard.

To experimentally confirm the *in silico* results, we carried out IEF siderotyping assays on representative strains of each of the clades defined in this work (except strains from Clade V that were not available in our lab). We found that strains belonging to one clade had similar pyoverdine production profiles and that the profile of strains within a clade was different from that of strains belonging to other clades (Figure 2).

Our bioinformatic and experimental results suggest that the average identity of genes forming part of the pyoverdine locus, together with bioinformatics using the predictor NRPSsp, can easily discriminate the type of pyoverdine produced by the different strains within the species *P. putida*, instead of more laborious techniques such as siderotyping.

### Pyoverdine production by clinical strains vs. environmental strains

*In silico* analysis of pyoverdine loci grouped the strains of *P. putida* HB13667 and HB3267 in Clade I with 98% sequence identity; both had been isolated from human blood. Although *P. putida* NBRC14164T was also grouped in Clade I, it belonged to a different subclade sharing a lower amino-acid identity (84%) with HB13667 and HB3267. *P. putida* H8234, H4184, and JCM18798 (with an overall identity of 86%) grouped into Clade II and were clearly separated from the other three clinical strains identity below 65% (Table 2). This suggests that siderophore-related proteins of the clinical isolates have at least two different phylogenic origins.

**Figure 3:**
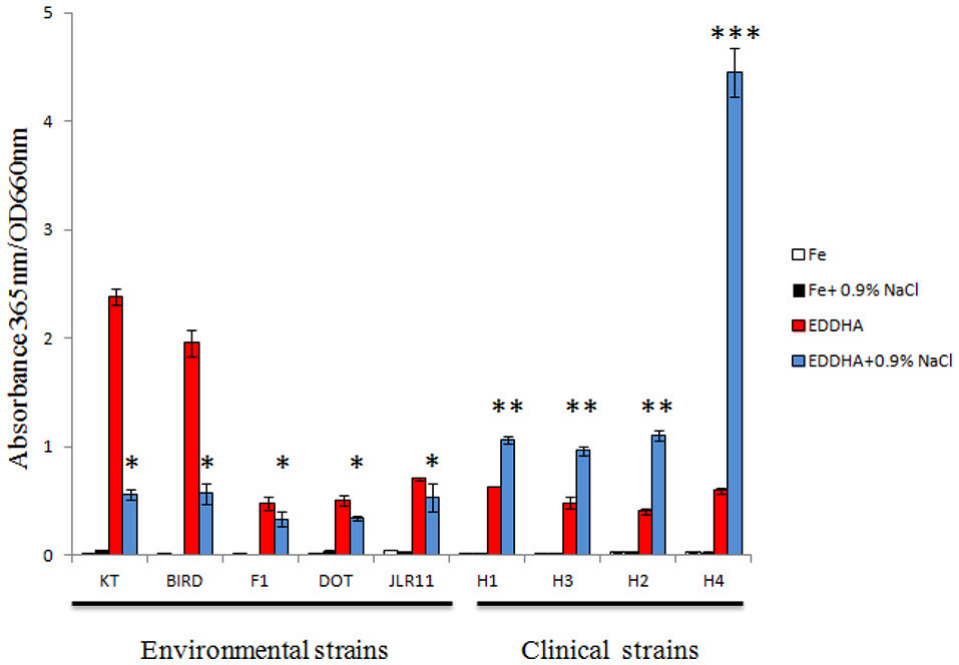
**Pyoverdine production by ***P. putida*** strains at low and a human serum osmolarity conditions**. KT, KT2440; DOT, DOT-T1E; H1, HB13667; H3, HB3267; H2, H8234; and H4, HB4184. Error bars mean standard deviation of 3 independent replicates. ^*^, ^**^, and ^***^ means statistically differenced groups (*p* < 0.01)

Interestingly, the pyoverdine-related proteins of isolate HB4184, isolated from human sputum, showed an identity close to 99.0% with *P. putida* B001 (Table 2), a rhizobacterium that was isolated from shore sand, oligotrophic and high salinity environment (Park et al., 2011; Table 2). To investigate if pyoverdine production in *P. putida* HB4184 was induced under high salt concentrations, we analyzed their production in a medium with the standard salt concentration used in laboratory media (0.05% w/v NaCl) and in the same medium but with 0.9% w/v NaCl content, in the presence or absence of the iron chelator EDDHA. Pyoverdine is secreted to the media in poor iron conditions; consequently, production of siderophores under iron rich conditions was barely perceptible in all the strains tested regardless of the salinity of the media (Figure 3). In the absence of free iron ions (in the presence of the iron chelator EDDHA) the production of a pyoverdine-type siderophore was increased in both media. This increase was especially significant in the environmental strains *P. putida* KT2440 and BIRD1 under standard laboratory medium. Production of pyoverdine was induced in the presence of high salt concentrations in all the clinical strains tested, but not in the environmental strains. This increase was significantly higher in strain *P. putida* HB4184 (Figure 3). This experiment suggests that clinical strains are better adapted to uptake iron under high salinity conditions than the environmental strains, with *P. putida* HB4184 being especially adapted to this condition.

### The diversity of the FpvA pyoverdine transporters in *P. putida*

Analysis of the homology amongst *P. putida* proteins involved in the synthesis and maturation of the chromophore part, in the transport of the peptidic part of pyoverdine or the complete siderophore, which are encoded by some genes of the pyoverdine locus, showed that they are highly conserved in all the *P. putida* studied strains (with an identity in the range between 75 and 90%). However, the identity percentage diminished when the pyoverdine receptor protein (FpvA) or the non-ribosomal peptide synthesis module PvdD, PvdJ, PvdI were analyzed (identity below 65% in most cases; data not shown). A similar observation was reported in *P. aeruginosa* strains by Smith et al. (2005). These authors proposed that the FpvA protein may be driving diversity at the pyoverdine locus. The outer membrane receptor FpvA, involved in the internalization of the pyoverdine-Fe complex following extracellular iron chelation, is specific for one pyoverdine type (Meyer et al., 2002; Smith et al., 2005; Nader et al., 2011). In *P. aeruginosa* three structurally different types of pyoverdine (types I, II, III) are produced, and each one is recognized at the outer membrane by the specific receptors, FpvAI, FpvAII, and FpvAIII, respectively (Cornelis et al., 1989).

**Figure 4:**
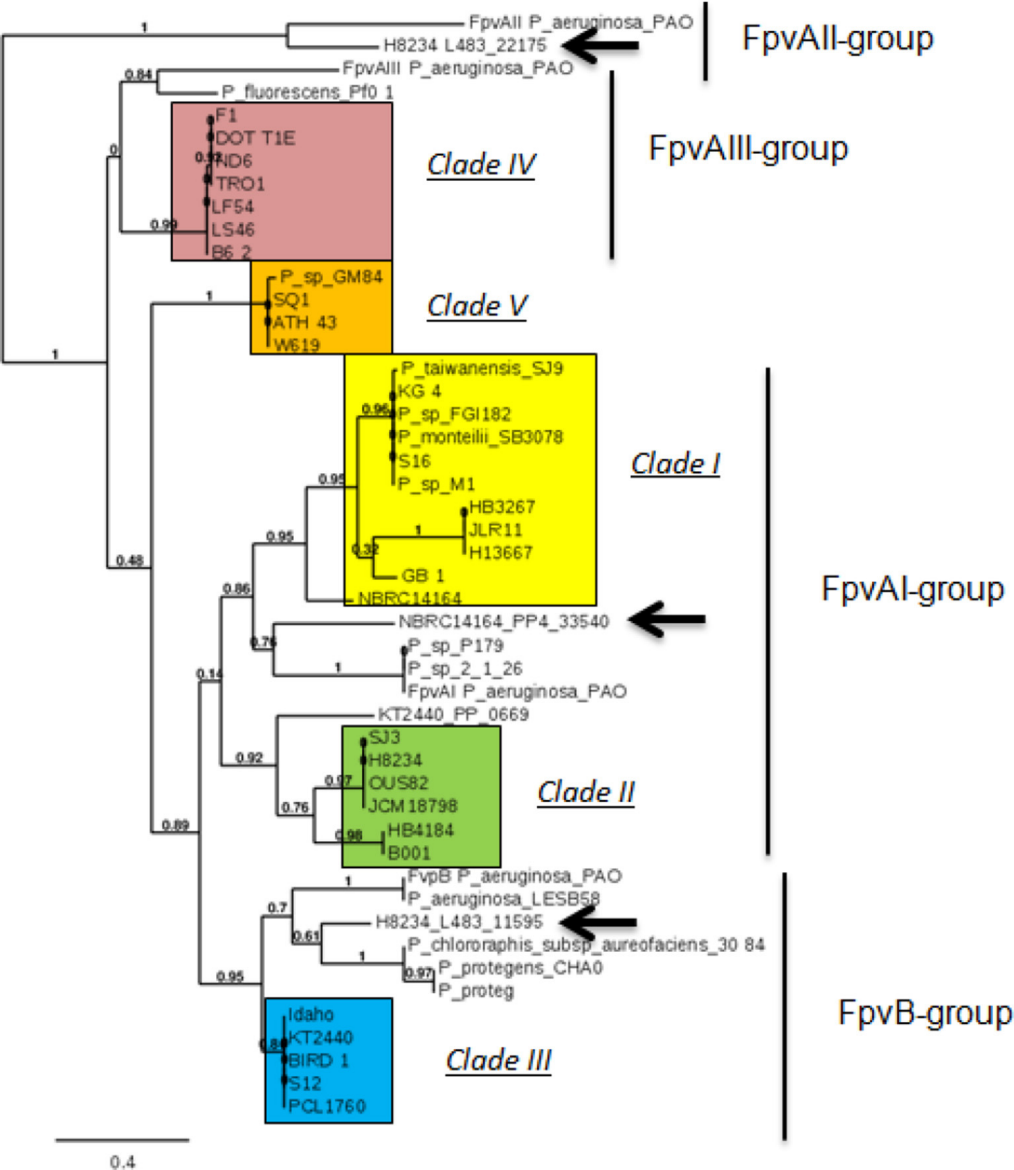
**Phylogenetic three of the FpvA proteins from different ***Pseudomonas*** strains**. Arrows represent exclusive TonB transporters from clinical strains. Clades are marked in the figure (square in yellow Clade I strains, in green Clade II strains, in blue Clade III strains, in red Clade IV strains, and in orange Clade V strains. Arrows indicate other TonB-dependent receptors from the studied strains that phylogenetically belong to the FpvA group. Software used to generate figure was Phylogeny.fr.

Classification of *P. putida* strains on the basis of the homology only amongst FpvA proteins gave a similar grouping of strains than when we used the complete pyoverdine locus (Table 1, Figure 4). Strains included in Clade I and II appear to belong to the classical FpvA receptor group (FpvA-I) where the FpvA type protein is similar to the FpvA of *P. aeruginosa* that recognizes the pyoverdine type I (Nader et al., 2011; Table 1, Figure 4). However, FpvA sequences of strains previously classified as Clade II are phylogenetically more distant to the sequences of strains belonging to Clade I; therefore we suggest that they may constitute different subgroups (Table 1, Figure 4).

Strains belonging to Clade III presented homology with an alternative receptor of type I pyoverdine named FpvB (Ghysels et al., 2004; Table 1, Figure 4). *P. putida* FpvA proteins belonging to Clade III are phylogenetically separated from other members of the group that are not *P. putida* species, such as *Pseudomonas protegens* Pf-5 (Hassan et al., 2010), *Pseudomonas chlororaphis* subsp. *aureofaciens* 30–84 (Loper et al., 2012), and *P. protegens* CHA0 (Ramette et al., 2011; Table 1, Figure 4). In the *P. putida* strains used in this study, the *fpvA* gene codifying the FpvB receptor is part of the pyoverdine locus (Figure 1) whilst in *P. aeruginosa* this gene is located elsewhere in the chromosome (Ghysels et al., 2004).

FpvA proteins of strains in Clade IV form a highly homogeneous group, which is related to the FpvA-III receptor from the opportunistic human pathogen *P. aeruginosa* PAO1 (Szmolka et al., 2012; Figure 4).

The FpvA protein of the endophitic strain W619 and strains SQ1 and ATH-43 (Clade V) form part of a different group of receptors, closer to that of *Pseudomonas* sp. GM84, a strain isolated from rhizosphere/endosphere of *Populus deltoides* (Brown et al., 2012).

Interestingly, *P. putida* JLR11, HB13667, HB3267 FpvA proteins were at least 98% identical, suggesting a common origin of this transporter in these three strains (Table 1, Figure 4). FpvA from HB4184 shows high homology (98%) with the rhizobacterium *P. putida* B001, isolated from a sandy shore soil from the Yellow Sea.

To sum up, the results described above indicate that phylogenetic studies based on the FpvA protein sequence of different *P. putida* strains yielded the same five clades that have been defined on the basis of overall protein homology with the pyoverdine-related genes (Table 1, Figure 4).

### Other siderophore transporters

*Pseudomonas* strains are also able to obtain iron through the acquisition of siderophores produced by other microorganisms (xenosiderophores) by TonB-dependent transporters located in their outer membrane (Schalk and Guillon, 2013). The function and number of TonB-dependent transporters in a particular strain have been related to their ability to adapt to changing environments and to colonize new niches (Cornelis, 2010; Llamas et al., 2014). TonB-dependent transporters are made up of three domains: the first one, a β-barrel with 11 large extracellular loops and periplasmic turns. The lumen of this barrel is occluded by a globular domain, which is the second domain, called the “plug” and the third domain consists of a long periplasmic N-terminal extension that is not involved in the transport of iron siderophores but participates in the regulation of transcription (Noinaj et al., 2010).

Analysis of the genome sequence of the *P. putida* strains revealed the presence of 7–14, depending on the strain, TonB-dependent transporters with these three domains, (Table 1, Supplementary Table 1). Remarkably, strains belonging to subclade A of Clade II and those belonging to subclade C or Clade I possess the highest number of these transporters (from 19 to 25) (Table 1, Supplementary Table 2). The presence of a higher number of TonB-dependent transporters than in the other *P. putida* strains seems to indicate that strains belonging to these subclades may have more versatility to acquire iron sources.

Most of the TonB-dependent transporters of the clinical strains *P. putida* HB13667, HB3267, and HB4184 are similar to those present in other *P. putida* strains (Supplementary Table 2), indicating than they are able to recognize the same type of xenosiderophores; this kind of transporter is considered to be non-exclusive. However, *P. putida* H8234 possesses two exclusive TonB-dependent transporters that have no homologs in any other *P. putida* strains we have studied. These transporters, namely L483_22175 and L483_11595, have their closest homologs in transporters identified in *P. aeruginosa* strains. L483-22175 presents 83% identity with *P. aeruginosa* isolates identified in cystic fibrosis patients (De Vos et al., 2001) and belongs to the FpvAII group (Figure 4) whilst L483_11595 belongs to the FpvB group (Figure 4) and its closest homolog (77% identity) was found in opportunistic human pathogens, i.e., *P. aeruginosa* strains PAO and LESB58. These results indicate that the *P. putida* isolate H8234 is able to recognize different siderophores from those recognized by other *P. putida* strains.

### Siderophore uptake by *P. putida* strains

To investigate the incorporation of different pyoverdines produced by several *Pseudomonas* strains, we measured the incorporation of *P. putida* siderophores bound with ^55^Fe into several *P. putida* strains. Siderophores produced by *P. putida* strains belonging to the same clade incorporated 100% of the complex siderophore-^55^Fe, with the only exception of *P. putida* HB4184 that was only able to uptake the 48.2% of siderophores produced by *P. putida* H8234. These two strains belong to different subclades, and differences at the level of pyoverdine-related proteins may account for this differential uptake. Cross-incorporation of siderophores between strains belonging to different clades was inferior to 4%.

As previously mentioned, the outer membrane TonB-dependent transporters are involved in the internalization of the xenosiderophore-Fe complexes and therefore we tested if siderophores produced by *P. aeruginosa* strains can be taken up by *P. putida* strains. In these assays we used the strains *P. aeruginosa* PAO1 that produces pyoverdine type I, *P. aeruginosa* 27,853 that produces pyoverdine type II, and *P. aeruginosa* Pa6 that produces pyoverdine type III. The level of siderophore (probably pyoverdine) cross-incorporation was below 10% in all cases, with the exception of *P. putida* DOT-T1E that was able to incorporate 18.8% of the siderophore-Fe complexes of *P. aeruginosa* strain PAO1, 21.7% of strain 27,853 and 73.8% of strain Pa6 (Table 3).

**Table 3:** **Cross-incorporation of pyoverdine between different ***Pseudomonas*** strains**.

	**Clade I**		**Clade II**		**Clade III**		**Clade IV**
**Strains**	**HB13667**	**HB3267**	**H8234**	**HB4184**	**KT2440**	**BIRD-1**	**DOT-T1E**
HB13667	100	100	0			0.7	0
H8234	2.8		100	48.2		1.2	0
KT2440					100	100	
DOT-T1E	3.5		0			1.3	100.0
PAO1	**6.1**	**1.5**	**7.9**	**9.1**		**4.1**	18.8
27853	0.3	0.4	1.5	0.4		2.1	21.7
Pa6	1.3	0	4.3	0		0	**73.8**

*Numbers refers to the mean of percentage of pyoverdine (Pvd) uptake compared to the incorporation of the native Pvd. In the first column, the strain source of pyoverdine, the second line the names of the uptaking strains. Highlighted in grey, maximum pyoverdine uptake; in bold, the highest values of siderophore from P. aeruginosa uptaken by P. putida strains (statistically differenced p < 0.01). These experiments were repeated at least twice, deviation errors were less than the 10% of the mean in all cases*.

In agreement with our phylogenetic studies of the FpvA protein, strains with FpvA proteins included in the FpvA-I and FpvB type of receptors (Figure 4), known to recognize pyoverdine type I (Cornelis et al., 1989; Ghysels et al., 2004) and belonging to Clade I, II, and III recognized the siderophores produced by the *P. aeruginosa* PAO1 (which mainly produces pyoverdine type I) better than siderophores produced by the other two *P. aeruginosa* strains (Table 3). Also in agreement with our classification, *P. putida* DOT-T1E (belonging to Clade IV and with FpvA protein included in the FpvA- III group; Figure 4), uptook siderophores more specifically produced by *P. aeruginosa* strain Pa6 (which mainly produces pyoverdine type III).

## Discussion

Traditionally the “core” genome of a species has been defined as those genes shared by all the strains of the species and amongst them, are included the majority of genes with housekeeping functions. These genes are interspersed with “accessory” genomic elements that are present in some but not all strains of the species (Kung et al., 2010). The accessory genome normally consists of integrative and conjugative elements, gene islands, prophages and phage-like elements, transposons, insertion sequences, and integrons (Mazel, 2016). The accessory genome of an individual strain is considered as an important driver of its ability to evolve and persist in a particular environment (Rodriguez-Valera et al., 2016). Therefore, niche adaptation is the result of interplay between “canonical” core genes and accessory genes. Interestingly, in *P. aeruginosa* the pyoverdine genes have been described as having a “core-accessory” character; they are present in all the strains that make up the species, but their codon usage, especially of the *fpvA* and the non-ribosomal peptide synthetase module genes, is different from the rest of the genes of the genome (Smith et al., 2005). This latter characteristic is specific to accessory genes, and is an indication of a differential evolution rate (Udaondo et al., 2016). This different codon usage is not consequence of direct acquisition from other microorganisms, but rather of the accumulation of polymorphisms and positive selection of these genes, which contribute to niche adaptation (Yang et al., 2012).

In this study we have classified the *P. putida* strains in five clades according to the global homology of the proteins codified by the pyoverdine loci. This distribution of strains in clades gave a similar but not identical phylogeny than that resulting from the basis of global genome phylogeny (Supplementary Figure 1) and the total proteome identity (Supplementary Table 1). Whilst the phylogeny based on pyoverdine locus, genome and proteome analysis is identical for all the isolates included in Clades II and V, genome and proteome phylogeny of strains included in Clades III and IV did not reveal the same pattern as the analysis with the pyoverdine locus (Supplementary Figure 1, Supplementary Table 1), suggesting a different evolution rate of the pyoverdine genes in these *P. putida* strains.

The high sequence homology of the protein encoded in the pyoverdine locus and the FpvA protein of the clinical isolates *P. putida* HB13667 and HB3267 (both isolated from blood of immune-depressed patients [Molina et al., 2016]), and the waste-water isolate JLR11 (included in Clade I, subclade A; Table 1), suggests that they either have a common ancestor or that the three strains have transited through similar environments and adapted to particular iron conditions. Waste water can be the equivalent to a laboratory for the fast evolution of bacteria: different bacterial strains coexist in these habitats and are exposed to pollutants coming from multiple human activities (agriculture, industry, and others). Bacterial survival under these conditions implies the acquisition of new capacities or the evolution of existing ones (Sharma et al., 2016). *P. putida* has an open-pangenome (Udaondo et al., 2016) and the acquisition of accessory genes and the evolution of those already existing in the core may help to colonize new environments. Gene acquisition from these niches has been demonstrated to be involved the capacity of some of *P. putida* strains to resist multiple antibiotics and the degradation of toxic compounds used in medicine, industry, and agriculture (Molina et al., 2013, 2014, 2016; Udaondo et al., 2016). However, the average total protein identity amongst the three strains is >95%, significantly higher than the identity with other strains of Clade I, thus reinforcing the idea of a common ancestor for the three strains.

The availability of iron is a frequent restrictive condition for the optimal growth of a microorganism in a given habitat and competition for its uptake is fierce in order to survive. The genomes of three of the clinical strains studied encode eight (*P. putida* HB13667 and HB3267) and ten (*P. putida* HB4184) TonB-dependent transporters related with xenosiderophore uptake that are similar to those identified in other environmental strains, suggesting that these strains acquire iron and perceive it in human fluids (from which they have been isolated) through uptake systems similar to those of environmental strains and that they have not acquired distinctive systems for the acquisition of xenosiderophores. Accordingly, their capacity to uptake siderophores from other *Pseudomonas* strains (i.e., *P. aeruginosa*) is quite limited (Table 3). The other three clinical strains *P. putida* H8234, NBRC14164T, and JCM18798 possess a high number of TonB-dependent transporters (19–20) (Table 1). These results suggest that these three clinical strains might be better suited for competing with other bacteria for iron scavenging. However, we did not observe significant differences in pyoverdine uptake in the different strains (Table 3), probably because these TonB-dependent transporters have not been related with pyoverdine uptake but rather with the uptake of different siderophores. Pyoverdine uptake has been related (Cornelis et al., 1989; Ghysels et al., 2004) with the type of FvpA-type therefore, strains belonging to Clade I and II uptake siderophores secreted by *P. aeruginosa* PAO1 that mainly produce pyoverdine type I, whilst *P. putida* DOT-T1E present the highest levels of uptake with siderophores produced by *P. aeruginosa* Pa6 (secreting mainly pyoverdine type III).

Pyoverdine-related genes systems of strain *P. putida* HB4184 are closely related to those of *P. putida* B001, a strain isolated from sandy shore soil and probably adapted to high salinity conditions. This similarity suggests that this clinical strain could be better adapted to uptake iron from environments with high osmotic pressure and this hypothesis was confirmed by the observation of a higher production of pyoverdine under iron deprivation conditions and in the presence of 0.9% of NaCl than the rest of the strains tested. *P. putida* HB4184 was isolated from sputum of an immuno-depressed patient with cystic fibrosis and although the normal concentrations of sodium and chloride ions in human saliva are lower than in serum, the concentrations of potassium, bicarbonate and iodine are higher. Furthermore, it has been reported than osmolarity in sputum from cystic fibrosis patients is higher than those in normal secretions, due to higher mucin concentrations in the secretions of these patients (Henderson et al., 2014). The induction of iron scavenging mechanisms under high salt conditions may be relevant under specific clinical conditions.

## Concluding remarks

The *P. putida* isolates which we studied can be classified into different clades according to the genetics of the siderophore biosynthesis and iron recycling (the identity of all the genes which make up the pyoverdine locus/i). In the case of the clinical strains, this classification suggests at least two possible different origins for these strains.

*P. putida* HB4184 (isolated from human sputum) is well adapted to uptake iron under high osmolarity conditions, a property that could be relevant for survival in specific human environments (such as sputum). *P. putida* H8234 and NBRC14164T possess unique TonB-dependent transporters, not found in any other clinical or environmental strains and therefore seem to be well adapted to compete with other microorganisms for iron scavenging.

The different phylogenetic origins of the pyoverdine locus in the *P. putida* clinical strains and the peculiarities of some of these strains suggest that either the clinical environment does not represent a high selective pressure in terms of iron scavenge and that the human environment is very diverse and adaptation of the different strains is mainly driven by these different conditions, or that *P. putida* strains are newcomers in hospital environments and that the iron-scavenge systems have had not enough time to evolve common mechanisms.

## Author contributions

Conceived and designed the experiments: LM, VG, JR. Performed the experiments: LM, VG, ZU. Analyzed the data: LM, VG, ZU, AS, JR. Wrote the paper: LM, VG, AS, JR.

### Conflict of interest statement

The authors declare that the research was conducted in the absence of any commercial or financial relationships that could be construed as a potential conflict of interest.
