# Supplementary material for: Iron Uptake Analysis in a Set of Clinical Isolates of Pseudomonas putida
Source: Front Microbiol. 2016 Dec 27;7:2100. doi: 10.3389/fmicb.2016.02100 (PMC5187384; doi:10.3389/fmicb.2016.02100)
Supplement: Supplementary file 1 [file Table1.DOCX]

Supp. Table 1. Average of the global identity of the proteins encoded by the different *P. putida* strains

| **Strain** | **H1** | **H3** | **JL** | ***P. t*** | ***P. m*** | **S16** | **KG** | **NB** | **GB** | **H8** | **SJ3** | **OU** | **JC** | **H4** | **B0** | **KT** | **Id** | **BI** | **S12** | **PC** | **F1** | **ND** | **DO** | **TR** | **LS** | **W6** | **SQ1** | **AT** | ***P. e*** | ***P. p*** |
| --- | --- | --- | --- | --- | --- | --- | --- | --- | --- | --- | --- | --- | --- | --- | --- | --- | --- | --- | --- | --- | --- | --- | --- | --- | --- | --- | --- | --- | --- | --- |
| **H13667** | 100 | 86.4 | **81.3** | **81.6** | **82.9** | **82.0** | 78.2 | 73.4 | 74.6 | 76.1 | 66.2 | 74.7 | 73.3 | 76.2 | 73.2 | 73.5 | 73.0 | 71.7 | 74.2 | 73.3 | 74.0 | 74.4 | 74.2 | 74.9 | 73.3 | 69.2 | 64.4 | 69.7 | 62.4 | 66.5 |
| **HB3267** | **92.3** | **100** | **90.0** | **85.6** | **87.4** | **86.6** | **84.2** | 79.0 | 79.3 | 78.9 | 69.3 | 77.8 | 76.6 | 79.3 | 78.0 | 78.5 | 77.1 | 77.2 | 78.4 | 77.7 | 78.7 | 79.0 | 78.1 | 78.9 | 78.0 | 73.7 | 68.9 | 71.8 | 67.1 | 71.3 |
| **JLR11** | **81.3** | **97.3** | **100** | **87.8** | **89.3** | **89.3** | **87.5** | **81.1** | **80.5** | **80.8** | 71.4 | **80.3** | 78.8 | **81.1** | **80.6** | **80.6** | 79.6 | **80.3** | **81.7** | **80.6** | **80.2** | **81.7** | **80.6** | **81.0** | **80.6** | 75.2 | 71.8 | 74.0 | 69.7 | 73.9 |
| ***P. t*** | 75.0 | 73.4 | 69.8 | **100** | 76.6 | 76.8 | 72.2 | 65.6 | 66.1 | 68.2 | 61.0 | 68.4 | 69.1 | 67.1 | 65.1 | 66.1 | 67.4 | 65.7 | 66.9 | 66.3 | 66.0 | 67.4 | 67.7 | 70.2 | 66.4 | 61.4 | 58.1 | 61.5 | 56.6 | 59.7 |
| ***P. m*** | **88.9** | **87.1** | **82.8** | **89.4** | **100** | **92.8** | **83.9** | 78.7 | 79.8 | 80.1 | 70.8 | 79.9 | 78.6 | 79.6 | 78.8 | 79.3 | 77.8 | 77.3 | 79.1 | 78.6 | 79.3 | **80.5** | **80.9** | **80.5** | 78.1 | 73.7 | 69.3 | 72.3 | 67.0 | 71.9 |
| **S16** | **87.2** | **85.6** | 81.7 | **87.3** | **92.7** | **100** | **82.9** | 77.9 | 78.0 | 79.3 | 69.6 | 78.7 | 77.5 | 78.4 | 77.5 | 78.5 | 77.3 | 76.2 | 77.5 | 77.6 | 77.4 | 79.4 | 78.2 | 79.4 | 77.2 | 72.5 | 68.9 | 72.1 | 66.4 | 71.6 |
| **KG** | **86.8** | **87.0** | **83.8** | **85.1** | **86.6** | **86.3** | **100** | **81.0** | 79.9 | 79.7 | 71.3 | 79.5 | 78.3 | **80.3** | **80.5** | 79.4 | 78.1 | 79.2 | 79.3 | **80.0** | 79.3 | **80.1** | **80.3** | 79.7 | 79.4 | 73.7 | 71.4 | 73.1 | 69.3 | 73.0 |
| **NB** | 78.1 | 78.0 | 75.3 | 76.3 | 77.9 | 77.5 | 78.1 | **100** | **82.5** | **85.4** | 76.7 | **85.1** | **83.3** | 79.8 | **80.5** | 78.9 | 77.8 | 78.8 | 79.3 | 79.0 | 78.8 | 79.9 | 79.8 | **80.4** | 79.9 | 72.7 | 69.9 | 72.1 | 67.8 | 71.8 |
| **GB-1** | 79.9 | 78.9 | 75.2 | 77.2 | 79.1 | 79.0 | 77.5 | **82.8** | **100** | **81.9** | **81.1** | **81.0** | **80.1** | **81.4** | **80.2** | **80.3** | 78.1 | 78.2 | 79.7 | 79.4 | **80.1** | **80.**4 | **80.0** | **80.4** | 79.5 | 73.2 | 68.9 | 71.7 | 67.8 | 72.6 |
| **H8234** | 72.1 | 70.2 | 66.7 | 71.4 | 72.0 | 71.9 | 68.5 | 76.1 | 73.2 | **100** | 77.5 | **91.7** | **89.3** | 73.6 | 73.1 | 71.8 | 76.2 | 71.0 | 72.6 | 72.9 | 72.3 | 73.3 | 73.1 | 75.8 | 71.4 | 68.0 | 62.5 | 66.6 | 59.5 | 63.0 |
| **SJ3** | 69.3 | 67.8 | 66.1 | 70.1 | 69.1 | 69.9 | 68.6 | 76.0 | 71.3 | **85.4** | **100** | **86.4** | **85.3** | 71.9 | 72.1 | 70.3 | 70.3 | 70.7 | 72.0 | 71.9 | 70.7 | 72.5 | 72.7 | 72.9 | 70.9 | 66.3 | 62.5 | 62.3 | 60.0 | 62.7 |
| **OUS82** | 70.1 | 67.6 | 64.6 | 69.3 | 70.0 | 69.1 | 66.7 | 74.6 | 70.6 | **90.6** | 76.5 | **100** | **95.1** | 71.2 | 71.2 | 69.5 | 71.5 | 69.3 | 71.0 | 69.6 | 70.1 | 72.0 | 72.4 | 75.6 | 69.6 | 66.6 | 61.8 | 65.0 | 58.6 | 61.9 |
| **JC** | 61.8 | 60.0 | 57.1 | 61.1 | 61.7 | 61.2 | 59.3 | 66.6 | 63.4 | **80.1** | 69.9 | **87.3** | **100** | 63.2 | 63.6 | 62.2 | 64.1 | 63.2 | 64.5 | 63.6 | 62.7 | 65.6 | 66.2 | 68.4 | 63.6 | 58.8 | 55.1 | 59.3 | 53.7 | 56.1 |
| **HB4184** | 79.2 | 77.8 | 73,7 | 76.7 | 78.1 | 77.6 | 76.0 | 78.8 | 79.9 | **80.1** | 71.4 | 79.4 | 78.4 | **100** | **85.5** | 77.9 | 76.4 | 76.7 | 78.0 | 77.6 | 78.0 | 79.8 | 78.4 | 78.2 | 77.6 | 72.0 | 67.2 | 64.1 | 65.1 | 69.5 |
| **B001** | 77.4 | 77.0 | 73.9 | 75.8 | 77.6 | 78.0 | 77.6 | **80.1** | 79.3 | **81.0** | 71.6 | **80.6** | 79.5 | **86.4** | **100** | 77.2 | 77.0 | 78.1 | 78.9 | 78.0 | 78.4 | 79.8 | 79.5 | 78.9 | 78.0 | 70.9 | 68.2 | 70.3 | 65.8 | 69.8 |
| **KT2440** | 77.4 | 76.7 | 73.2 | 75.5 | 77.4 | 77.8 | 74.4 | 77.6 | 78.8 | 78.5 | 70.3 | 77.9 | 76.9 | 78.0 | 76.3 | **100** | **84.6** | **82.6** | **84.0** | **83.1** | **84.3** | **85.4** | **84.6** | **84.4** | **84.5** | 72.8 | 68.2 | 71.0 | 65.1 | 68.9 |
| **Idaho*** | 69.4 | 68.4 | 65.4 | 69.1 | 68.6 | 68.3 | 66.9 | 69.1 | 69.3 | 71.9 | 62.9 | 71.3 | 70.5 | 69.5 | 68.8 | 76.9 | **100** | 75.5 | 78.3 | 76.0 | 74.8 | 76.9 | 76.5 | 78.1 | 75.3 | 64.3 | 61.4 | 63.9 | 57.8 | 61.6 |
| **BIRD-1** | 79.1 | 78.6 | 75.7 | 77.0 | 79.1 | 78.6 | 78.6 | **81.2** | **80.3** | **81.5** | 72.7 | **80.4** | 79.2 | **80.4** | **80.5** | **86.3** | **85.8** | **100** | **94.3** | **93.9** | **85.7** | **89.2** | **88.6** | **87.0** | **87.5** | 73.6 | 70.6 | 72.6 | 67.2 | 71.4 |
| **S12*** | 74.8 | 73.8 | 72.3 | 70.4 | 73.9 | 73.1 | 72.6 | 75.0 | 75.1 | 77.0 | 68.6 | 76.1 | 74.8 | 75.2 | 74.8 | **81.1** | **83.2** | **86.6** | **100** | **88.3** | **81.6** | **82.6** | **82.3** | **81.7** | **81.2** | 69.8 | 66.0 | 68.6 | 62.5 | 66.3 |
| **PCL1760** | 77.8 | 76.6 | 73.8 | 75.7 | 77.6 | 77.1 | 76.2 | 78.4 | 78.5 | **80.5** | 71.2 | 79.6 | 78.5 | 78.4 | 79.6 | **84.1** | **84.6** | **91.5** | **93.0** | **100** | **85.9** | **87.0** | **87.2** | **86.0** | **85.2** | 72.2 | 69.0 | 71.5 | 65.2 | 69.1 |
| **F1** | **81.2** | **80.0** | 76.2 | 78.7 | **81.1** | 79.9 | 78.5 | **81.2** | 82.0 | **82.0** | 73.5 | **81.2** | **80.3** | **81.6** | **81.0** | **88.3** | **86.0** | **87.0** | **88.1** | **88.4** | **100** | **92.6** | **91.1** | **90.0** | **88.8** | 76.1 | 71.1 | 74.1 | 67.8 | 71.2 |
| **ND6*** | 77.8 | 77.0 | 73.9 | 76.2 | 78.5 | 78.0 | 75.9 | 78.3 | 78.8 | 79.8 | 71.2 | 79.3 | 78.2 | 79.7 | 78.5 | **85.7** | **84.5** | **84.8** | **85.5** | **85.6** | **88.8** | **100** | **89.8** | **88.0** | **87.5** | 73.1 | 68.5 | 71.4 | 65.3 | 70.1 |
| **DOT** | 77.0 | 75.5 | 71.6 | 74.7 | 78.0 | 75.9 | 74.4 | 77.7 | 77.7 | 79.1 | 70.0 | 77.9 | 76.9 | 77.6 | 76.9 | **83.1** | **82.4** | **82.7** | **83.7** | **84.3** | **86.0** | **88.2** | **100** | **87.4** | **86.3** | 71.5 | 67.4 | 70.4 | 63.8 | 67.8 |
| **TRO1*** | 73.2 | 72.1 | 68.4 | 74.1 | 73.0 | 72.3 | 70.2 | 73.6 | 73.5 | 77.2 | 67.0 | 78.1 | 77.2 | 73.8 | 72.5 | 78.8 | **80.1** | 77.5 | 79.1 | 78.9 | **80.5** | **82.0** | **82.9** | **100** | **84.7** | 68.1 | 63.9 | 67.7 | 60.8 | 64.6 |
| **LS46*** | 78.1 | 77.3 | 74.2 | 76.9 | 77.4 | 77.1 | 76.3 | 79.8 | 79.1 | 79.5 | 71.1 | 78.3 | 77.7 | 79.1 | 78.1 | **85.9** | **84.4** | **84.7** | **85.8** | **85.8** | **87.0** | **89.2** | **89.7** | **88.1** | **100** | 71.2 | 69.1 | 72.0 | 66.1 | 70.0 |
| **W619** | 77.0 | 75.7 | 72.2 | 74.2 | 76.2 | 75.6 | 73.8 | 75.7 | 74.6 | 77.7 | 69.8 | 77.8 | 76.9 | 76.2 | 74.2 | 76.4 | 74.8 | 74.1 | 76.4 | 75.7 | 76.6 | 77.1 | 76.3 | 76.8 | 75.2 | **100** | **83.6** | **85.8** | 68.5 | 73.1 |
| **SQ1** | 65.0 | ¡63.6 | 64.1 | 70.2 | 71.8 | 71.7 | 71.4 | 72.7 | 71.3 | 73.2 | 65.5 | 73.0 | 71.5 | 71.6 | 71.4 | 72.6 | 71.5 | 71.8 | 72.4 | 72.4 | 72.2 | 72.6 | 72.8 | 72.5 | 72.1 | **84.9** | **100** | **84.6** | 66.1 | 71.2 |
| **ATH-43** | 70.1 | 68.2 | 65.2 | 67.8 | 68.3 | 68.3 | 67.1 | 68.7 | 68.0 | 70.7 | 60.3 | 69.6 | 69.2 | 69.0 | 67.2 | 69.1 | 68.1 | 67.7 | 69.3 | 68.5 | 68.9 | 69.4 | 69.5 | 70.1 | 68.8 | 79.4 | **76.8** | **100** | 62.1 | 67.1 |

**H1-H13667, H3- HB3267, JL-JLR11, P.t- *P. taiwanensis, P.m- P. monteilli,* GB-GB-1, NB-NBRC 14164, KG-KG-4, H8-H8234,OU- OUS82, JC- JCM 18798, H4- HB4184, B0- B001, KT-KT2440, Id-Idaho, BI-BIRD-1, PC-PCL1760, ND-ND-6, DO-DOT-T1E, TR-TRO1, LS- LS46, W6-W619, AT- ATH-43, P.e- *P. entomophila, P.p- P. plecoglossicida.* Numbers represent the % of identity in protein identity, in bold values over 80%. Highlighted in yellow, green , blue, red and orange identity values over 80% with strains Clade I, Clade II, Clade III, Clade IV and Clade V respectively, dark tonalities when the identity values were over 82.5%.**
