# Supplementary material for: Iron Uptake Analysis in a Set of Clinical Isolates of Pseudomonas putida
Source: Front Microbiol. 2016 Dec 27;7:2100. doi: 10.3389/fmicb.2016.02100 (PMC5187384; doi:10.3389/fmicb.2016.02100)
Supplement: Supplementary file 2 [file Table2.DOCX]

Suppl. Table 2. Other siderophore TonB-dependent transporters, different that FpvA placed in the pyoverdine locus/i, from the studied *P. putida* strains which have classical structure (TonB N-terminal locus, plug and barrel)

|  | **H13667** | **HB3267** | ***P_monteilii*** | **S16** | **NBRC14164** | **GB-1** | **H8234** | **HB4184** | **KT2440** | **BIRD-1** | **F1** | **DOT-T1E** | **ND6** | **W619** |
| --- | --- | --- | --- | --- | --- | --- | --- | --- | --- | --- | --- | --- | --- | --- |
| Achromobactin receptor | ORF4840 | B479_15270 | X970_14390 | PPS_3070 | PP4_22510 | PputGB1_2339 | L483_18745 | ORF2721 | PP_3575 | PPUBIRD1_2217 | Pput_2196 | T1E_2286 | YSA_00173 | - |
| Iron siderophore receptor | ORF2552 | B479_22295 | X970_21465 | PPS_4432 | PP4_07710 | PputGB1_4588 | L483_27370 | ORF2566 | PP_4606 | PPUBIRD1_4301 | Pput_4465 | T1E_1570 | YSA_03151 | PputW619_0858 |
| Aerobactin receptor | ORF5099 | B479_08910 | X970_06830 | PPS_1804 | PP4_36230 | PputGB1_1817 | L483_08760 | ORF1200 | PP_2193 | PPUBIRD1_3458 | Pput_3544 | T1E_1276 | YSA_01391 | - |
| FecA-like | ORF3601 | B479_04835 | X970_02885 | PPS_0934 | PP4_44060 | PputGB1_0911 | L483_04390 | ORF3272 | PP_0867 | PPUBIRD1_0918 | Pput_0897 | T1E_5136 | YSA_06818 | PputW619_4311 |
| TonB-dependent siderophore receptor | - | - | - | - | - | PputGB1_0378 | L483_01790 |  | PP_0350 | PPUBIRD1_0385 | Pput_0376 | T1E_2635 | YSA_05714 | PputW619_4852 |
| TonB-dependent siderophore receptor | - | - | - | - | PP4_24380 | PputGB1_2781 | - | - | PP_3084 | - | - | - | - | - |
| Ferrioxamine B receptor | ORF2874 | B479_01140 | X970_26500 | PPS_0127 | PP4_01540 | PputGB1_0178 | L483_00475 | ORF5422 | PP_0160 | PPUBIRD1_0190 | Pput_0179 | T1E_0094 | YSA_05305 | - |
| TonB-dependent siderophore receptor | - | - | - | - | PP4_47320 | PputGB1_4761 | L483_28640 | ORF0396 | PP_4755 | PPUBIRD1_4461 | Pput_4624 | T1E_0682 | YSA_03595 | - |
| FecA, iron(III) dicitrate receptor | - | - | - | - | PP4_07630 | PputGB1_4596 | L483_27410 | - | PP_4613 | - | - | - | - | - |
| Ferric siderophore receptor | - | - | - | - | - | - | - | - | PP_0669 | - | - | - | - | - |
| Ferric-pseudobactin M114 receptor | - | - | - | - | PP4_37750 | PputGB1_1585 | L483_07615 | ORF3744 | - | PPUBIRD1_3580 | Pput_3666 | T1E_0064 | YSA_01637 | - |
| TonB-dependent siderophore receptor | - | - | - | - | PP4_33820 | PputGB1_2065 | L483_09895 | - | - | PPUBIRD1_3262 | - | - | - | - |
| TonB-dependent siderophore receptor | - | - | - | - | - | - | - | - | - | PPUBIRD1_3161 | Pput_3196 | T1E_3391 | YSA_00523 | - |
| Putative siderophore receptor | ORF5617 | B479_11795 | X970_09485 | PPS_2296 | - | - | - | ORF2936 | - | PPUBIRD1_2697 | - | - | - | - |
| Heme receptor | ORF0169 | B479_05310 | X970_03370 | PPS_1035 | PP4_43100 | PputGB1_1005 | L483_04880 | ORF3174 | PP_1006 | PPUBIRD1_1056 | Pput_1043 | T1E_1983 | YSA_07092 | PputW619_4218 |
| TonB-dependent siderophore receptor | - | - | - | - | - | PputGB1_0860 | - | - | - | PPUBIRD1_0868 | Pput_0846 | T1E_4604 | YSA_06718 | - |
| TonB-dependent siderophore receptor | - | - | - | - | - | PputGB1_4072 | - | - | - | - | - | - | - | - |
| fpvA, ferripyoverdine receptor | - | - | - | - | - | PputGB1_3327 | - | - | - | - | - | - | - | - |
| ferrioxamine receptor | - | - | - | - | PP4_33070 | PputGB1_3322 | L483_10205 | - | - | - | - | - | - | - |
| ferripyoverdine receptor FpvA | - | - | - | - | PP4_33040 | PputGB1_3317 | L483_10215 | - | - | - | - | - | - | - |
| TonB-dependent siderophore receptor | - | - | - | - | - | PputGB1_3280 | - | - | - | - | - | - | - | - |
| TonB-dependent siderophore receptor | - | - | - | - | - | PputGB1_0759 | - | - | - | - | - | - | - | - |
| TonB-dependent siderophore receptor | - | - | - | - | - | PputGB1_0747 | - | - |  | - | - | - | - | - |
| TonB-dependent siderophore receptor | - | - | - | - | - | PputGB1_0693 | - | - | - | - | - | - | - | - |
| TonB-dependent siderophore receptor | - | - | - | - | - | PputGB1_0692 | - | - | - | - | - | - | - | - |
| TonB-dependent siderophore receptor | - | - | - | - | - | PputGB1_3358 | - | - | - | - | - | - | - | - |
| TonB-dependent siderophore receptor | - | - | - | - | - | PputGB1_0701 | - | - | - | - | - | - | - | - |
| TonB-dependent siderophore receptor | - | - | - | - | - | - | - | - | - | - | - | - | - | PputW619_4481 |
| TonB-dependent siderophore receptor | - | - | - | - | - | - | - | - | - | - | - | - | - | PputW619_3478 |
| TonB-dependent siderophore receptor | ORF5216 | B479_02230 | X970_00055 | PPS_0346 | PP4_03810 | - | - | ORF0859 | - | - | - | - | - | - |
| TonB-dependent siderophore receptor | - | - | - | - | - | - | L483_16550 | - | - | - | - | - | - | - |
| TonB-dependent siderophore receptor | - | - | - | - | PP4_32030 | - | L483_10610 | - | - | - | - | - | - | - |
| ferrioxamine receptor | - | - | - | - | PP4_11870 | - | L483_25170 | - | - | - | - | - | - | - |
| ferripyoverdine receptor FpvA | - | - | - | - | PP4_28380 | - | L483_13500 | - | - | - | - | - | - | - |
| Enantiopyochelin Tonb-Dependent Transporter | - | - | - | - | - | - | L483_22175 | - | - | - | - | - | - | - |
| second ferric pyoverdine receptor FpvB | - | - | - | - | - | - | L483_11595 | - | - | - | - | - | - | - |
| ferripyoverdine receptor FpvA | - | - | - | - | PP4_33540 | - | - | - | - | - | - | - | - | - |
| putative TonB-dependent receptor | - | - | - | - | PP4_06080 | - | - | - | - | - | - | - | - | - |
| **TOTAL** | **8** | **8** | **8** | **8** | **20** | **24** | **19** | **10** | **11** | **13** | **11** | **11** | **11** | **6** |
