# Supplementary figures and images for: Iron Uptake Analysis in a Set of Clinical Isolates of Pseudomonas putida
Source: Front Microbiol. 2016 Dec 27;7:2100. doi: 10.3389/fmicb.2016.02100 (PMC5187384; doi:10.3389/fmicb.2016.02100)

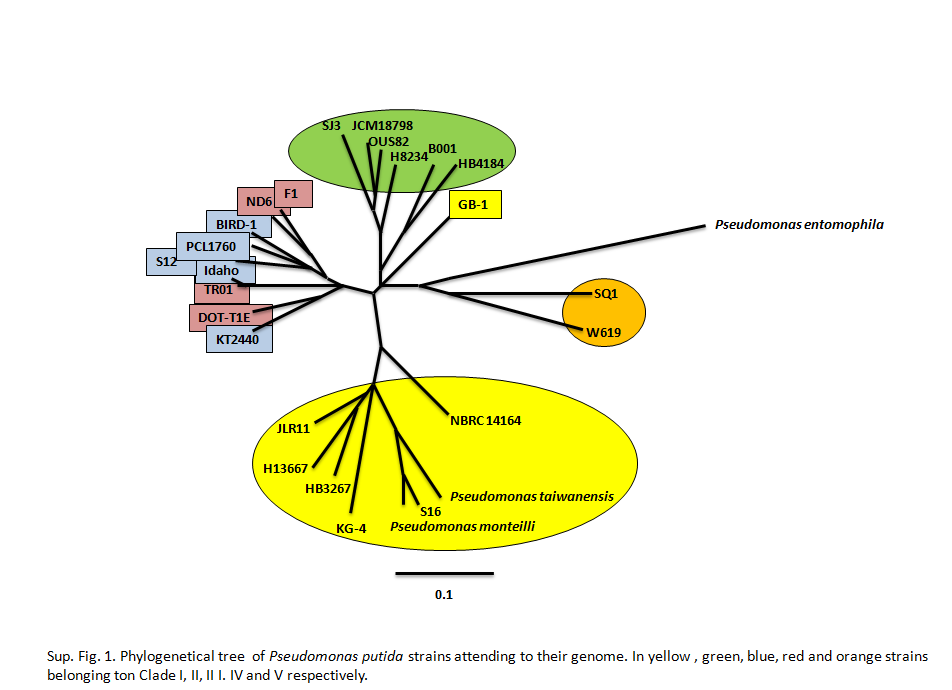

Supplement: Supplementary file 3 [file Image1.TIF]
